# Multimodal Neuroprotection in Ischemic Stroke: Emerging Non-Pharmacological Interventions from Bench to Bedside

**DOI:** 10.3390/brainsci15101111

**Published:** 2025-10-15

**Authors:** Junzhao Cui, Jingyi Yang, Luji Liu, Xiaoyun Liu, Xunming Ji

**Affiliations:** 1 Department of Neurology, The Second Hospital of Hebei Medical University, Shijiazhuang 050000, China; cuijunzhao@hebmu.edu.cn (J.C.); liuluji140018@126.com (L.L.); 2 Department of Data Center, The Second Hospital of Hebei Medical University, Shijiazhuang 050000, China; yangjingyi@hebmu.edu.cn; 3 Department of Neurology, The First Hospital of Hebei Medical University, 89 Donggang Road, Yuhua District, Shijiazhuang 050000, China; 4 Department of Neurosurgery, Xuanwu Hospital, Capital Medical University, No. 45, Changchun Street, Xicheng District, Beijing 100053, China

**Keywords:** neuroprotection, hypothermia, remote ischemic conditioning, normobaric hyperoxia

## Abstract

Currently, the effective therapeutic strategies for acute ischemic stroke (AIS) remain revascularization therapies, including intravenous thrombolysis and endovascular thrombectomy. However, the narrow time window and reperfusion injury associated with reperfusion therapy limit favorable outcomes in some patients. As adjuncts to revascularization, certain neuroprotective agents have demonstrated robust preclinical results, but only a few have achieved successful clinical translation due to challenges in dosing and safety concerns. In recent years, convenient and relatively safe non-pharmacological neuroprotective interventions—such as hypothermia, remote ischemic conditioning (RIC), and normobaric hyperoxia (NBO)—have gained increasing research attention. These approaches offer advantages including high safety profiles, excellent tolerability, low cost, and the potential to synergize with reperfusion therapy, underscoring their broad clinical applicability. Numerous clinical trials have validated their potential to improve neurological functional outcomes, and this review explores the mechanisms and clinical applications of non-pharmacological neuroprotective therapies in ischemic stroke.

## 1. Introduction

Ischemic stroke remains one of the leading global causes of mortality and long-term disability, with persistently high incidence and debilitating rates [1]. Although thrombolytic therapy is currently the only acute ischemic stroke treatment approved by the U.S. Food and Drug Administration (FDA), its clinical application remains restricted, with a significant number of patients precluded from therapeutic benefits due to the narrow treatment time window and stringent inclusion criteria [2,3,4]. Furthermore, while reperfusion therapies have significantly improved vascular recanalization rates, subsequent reperfusion injury and inflammatory cascade responses continue to adversely affect patient prognosis [5]. Consequently, developing novel neuroprotective strategies to mitigate ischemic brain injury has emerged as a critical research focus in neuroscience, with particular emphasis on creating complementary approaches that synergize with successful recanalization to enhance clinical outcomes. Over past decades, numerous neuroprotective agents have been extensively investigated in preclinical stroke models [6,7]. However, a notable translational gap persists, as compounds demonstrating robust efficacy in experimental settings frequently fail to achieve the expected therapeutic benefits in clinical trials, posing substantial challenges for clinical implementation of neuroprotective interventions. Additionally, compounded by high research expenditures and diminishing translational returns, investment in this field has progressively declined. Against this backdrop, exploring innovative protective mechanisms for stroke management has become an urgent priority.

**Figure 1:**
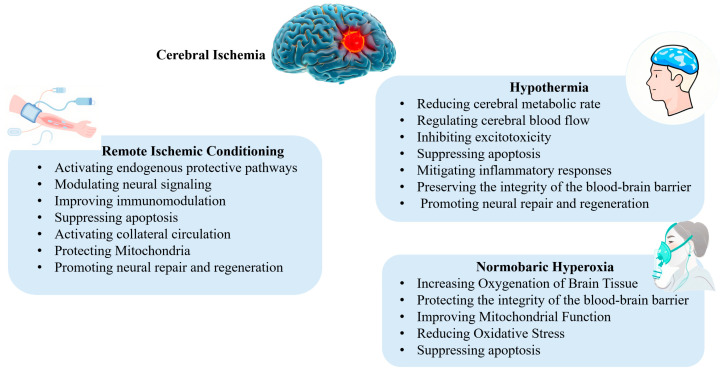
The non-pharmacological neuroprotective mechanisms for acute ischemic stroke.

Non-pharmacological interventions exhibit unique advantages through their multi-target modulatory properties, enabling precise interference with the pathophysiological cascade of acute ischemic stroke. These approaches effectively contain infarct expansion while preserving the ischemic penumbra [8,9,10]. Distinct from conventional neuroprotective pharmacotherapies that rely on systemic circulation for delivery, physical neuroprotection technologies demonstrate breakthrough potential by overcoming the dependency on blood perfusion [11]. This review systematically reviews several promising neuroprotective strategies with potential for clinical application, which offer novel perspectives for improving functional recovery and long-term prognosis in patients with AIS. The non-pharmacological neuroprotective mechanisms for acute ischemic stroke are shown in Figure 1.

## 2. Methods

We conducted a systematic review following the Preferred Reporting Items for Systematic Reviews and Meta-Analyses (PRISMA) guidelines [12].

### 2.1. Search Strategy

We conducted a systematic review following the Preferred Reporting Items for Systematic Reviews and Meta-Analyses (PRISMA) guidelines. We searched PubMed, Embase and Web of Science for English-language clinical studies and reviews published from database inception through May 2025. The search strategy incorporated keywords including “ischemic stroke” AND (“therapeutic hypothermia” OR “cooling” OR “remote ischemic conditioning” OR “normobaric hyperoxia”). Duplicate records were removed. Additionally, we manually screened reference lists of key articles to identify additional relevant publications.

### 2.2. Selection Process

**Figure 2:**
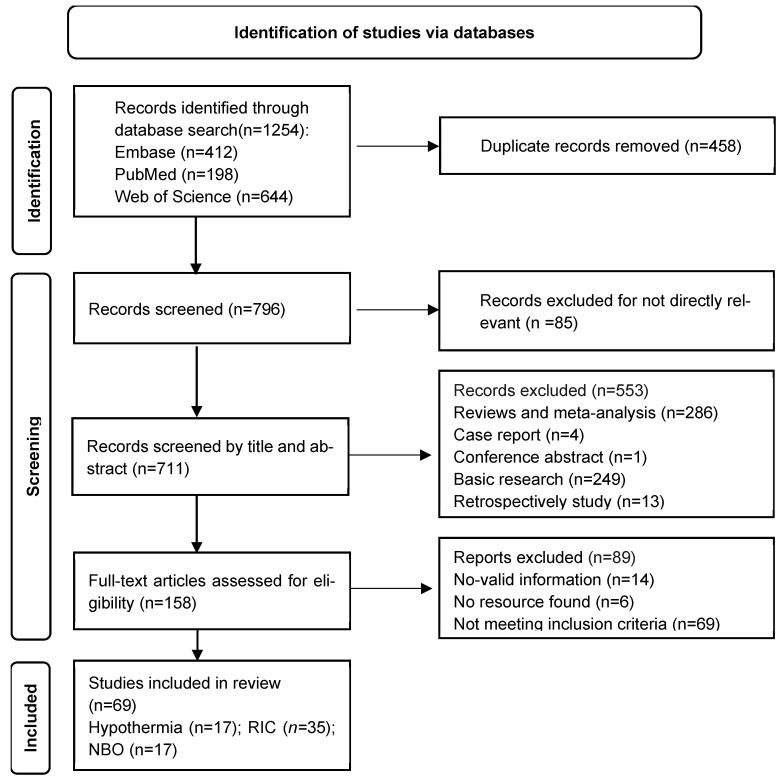
PRISMA Flow Diagram. PRISMA, Preferred Reporting Items for Systematic Reviews and Meta-Analyses; RIC, Remote Ischemic Conditioning; NBO, Normobaric hyperoxia.

Two authors (J.Z.C. and L.J.L.) independently screened the articles based on titles and abstracts. Any discrepancies were resolved by a third author (X.Y.L.). Studies were included in the systematic review according to the following predetermined criteria: (1) clinical studies; (2) patients with ischemic stroke; (3) the intervention group received therapeutic hypothermia, remote ischemic conditioning, or normobaric hyperoxia. Retrospective studies were excluded. The study selection process is illustrated in Figure 2.

### 2.3. Assessment of Bias

The risk of bias in the included studies was assessed by three independent reviewers (J.Z.C., J.Y.Y., and L.J.L.) using the Cochrane risk assessment tool. The following domains of bias were evaluated: selection bias, performance bias, detection bias and other potential sources of bias. The studies were categorized as having a low, unclear, or high risk of bias. Any disagreements among the reviewers were resolved by consulting a fourth reviewer (X.Y.L.).

## 3. Hypothermia

Therapeutic hypothermia has been recognized as an effective neuroprotective agent in experimental stroke models and stroke patients, extending the therapeutic time window for other neuroprotective strategies [11,13,14,15]. As a neuroprotective approach, hypothermia demonstrates unique advantages through multiple mechanisms, including reducing metabolic rate, inhibiting oxidative stress, and suppressing apoptosis. Hypothermia has achieved significant success in the treatment of brain injury after cardiac arrest and neonatal hypoxic–ischemic encephalopathy [16] and has been incorporated into clinical guidelines. However, the translation of hypothermic therapy into acute ischemic stroke (AIS) treatment still faces numerous challenges. Although animal experiments and small-scale clinical studies have shown that hypothermia has potential neuroprotective effects, its efficacy has not been consistently confirmed in large-scale clinical trials. To optimize the neuroprotective benefits of hypothermia, researchers are exploring a variety of optimization strategies. On the one hand, improvements in cooling methods—such as selective brain cooling and endovascular cooling [17,18,19,20] aim to mitigate systemic side effects. On the other hand, combining hypothermia with other neuroprotective agents or therapies (such as thrombolysis, anti-inflammatory drugs, antioxidants) [21,22,23] may enhance its efficacy. Furthermore, a deeper understanding of the molecular mechanisms of hypothermia, such as its effects on apoptotic pathways, inflammatory signaling, and neuro regeneration, will provide a more robust theoretical foundation for clinical applications.

### 3.1. Mechanisms of Hypothermia-Induced Neuroprotection

The neuroprotective effects of therapeutic hypothermia in ischemic brain injury are mediated through multiple mechanisms.

During the acute ischemic phase (lasting from minutes to hours), therapeutic hypothermia mediates neuroprotective mechanisms through three principal pathways: metabolic regulation, cerebral hemodynamic modulation, and excitotoxic cascade attenuation. Notably, hypothermia induces reduction in cerebral metabolic rate, demonstrating approximately 5–7% decrement per 1 °C temperature decrease. This metabolic suppression preserves cellular energy homeostasis through a cascade of protective effects: (1) attenuated ATP consumption reduces oxygen-glucose demand; (2) diminished anaerobic glycolysis subsequently decreases lactic acid accumulation; and (3) prevention of intracellular pH imbalance mitigates acidosis-mediated neuronal injury [24,25]. The effects of hypothermia on cerebral blood flow are more complex. In the absence of injury, hypothermia decreases cerebral blood flow, whereas after ischemia and reperfusion, hypothermia can mitigate the hyperemia and subsequent reduction in cerebral blood flow [24]. Additionally, hypothermia can improve microcirculation by reducing microvascular constriction [26]. Hypothermia also reduces excitotoxic injury by decreasing the release and accumulation of excitatory amino acids (such as glutamate). One study reveals that hypothermia inhibits the degradation of the glutamate receptor 2 (GluR2) subunit of the AMPA receptor, effectively preventing pathological calcium influx and subsequent neuronal apoptosis [27].

During the subacute phase of ischemia (1–7 days), hypothermia exerts neuroprotective effects by suppressing apoptosis, mitigating inflammatory responses, and preserving blood–brain barrier integrity. Hypothermia modulates multiple apoptotic pathways: in the intrinsic pathway, it regulates expression of BCL-2 family members, reduces cytochrome c release, and inhibits caspase activation [28]; in the extrinsic pathway, it suppresses FAS/FAS ligand (FASL)expression and caspase-8 activation [29]. Additionally, hypothermia attenuates pro-apoptotic effects by inhibiting phosphatase and tensin homologue (PTEN) dephosphorylation [30]. An in-depth elucidation of these signaling pathways provides a critical foundation for understanding the mechanisms of non-pharmacological interventions. Previous studies have implicated that the inhibitor of apoptosis-stimulating protein of p53 (iASPP) may play a role in neuronal death after stroke [17]. Xiangrong Liu et al. [18], utilizing both in vitro (oxygen-glucose deprivation/reperfusion, OGD/R model) and in vivo (middle cerebral artery occlusion, MCAO mice) models, demonstrated that mild therapeutic hypothermia (33 °C) alleviates ischemia/reperfusion-induced neuronal apoptosis, mitigates cerebral injury, and improves neurological deficits in mice by upregulating iASPP expression while suppressing the activity of its pro-apoptotic targets, including Puma, Bax, and cleaved caspase-3. Crucially, the protective effects of hypothermia were abolished when iASPP expression was genetically inhibited, confirming iASPP as a critical endogenous mediator of hypothermia-induced neuroprotection. These findings highlight how a non-pharmacological physical intervention can harness intrinsic cellular pathways to achieve therapeutic effects. Future research should prioritize elucidating the precise molecular mechanisms by which non-pharmacological strategies like hypothermia regulate apoptosis networks, including the interaction of effectors like iASPP with p53 family members. Such mechanistic insights are crucial for advancing the development of targeted, non-pharmacological strategies against cerebral ischemia/reperfusion injury. Hypothermia suppresses inflammatory responses through multiple mechanisms, which involve reduced neutrophil infiltration and microglial activation, accompanied by decreased levels of inflammatory mediators including reactive oxygen species (ROS), reactive nitrogen species (RNS), adhesion molecules, and pro-inflammatory cytokines (e.g., IL-1β, TNF-α, IL-6) [19,20,31,32,33]. Hypothermia further inhibits nuclear factor κB (NF-κB) activation, thereby downregulating inflammation-related gene expression [34]. Furthermore, hypothermia preserves blood–brain barrier integrity by suppressing the activity of matrix metalloproteinases (MMPs), thereby reducing degradation of extracellular matrix components and tight junction proteins [35].

During the chronic phase of ischemia (weeks to months), hypothermia positively influences neural repair and regeneration. It enhances neurogenesis and synaptogenesis by promoting the proliferation and differentiation of neural stem cells [36]. Notably, a study by Kurisu et al. demonstrated that hypothermia prevents post-ischemic reactive gliosis and glial scar formation [37]. However, the mechanisms by which hypothermia affects gliogenesis require further investigation.

In summary, hypothermia-mediated neuroprotection in cerebral ischemia involves multifaceted mechanisms, with its multi-target nature positioning it as a highly promising therapeutic strategy [38]. Nevertheless, clinical translation faces challenges, including the need to optimize treatment protocols, minimize adverse effects, and identify patient populations most likely to benefit from this intervention.

### 3.2. Methods of Hypothermia Induction

The application of hypothermia therapy in stroke management has undergone a significant evolution from traditional whole body cooling hypothermia to localized inducing hypothermia. Conventional whole body hypothermia primarily relied on systemic cooling through surface methods (e.g., cooling blankets, ice packs) or intravenous cooling, yet its utility was constrained by prolonged induction times and systemic adverse effects such as cardiac arrhythmias and platelet aggregation [39,40,41,42]. To enhance targeting precision, research shifted toward localized hypothermic strategies, including selective head cooling (e.g., cooling helmets, nasopharyngeal cooling) [15,43] and intra-arterial local cooling (IACI) [10,44,45,46,47,48,49,50]. Preclinical studies have demonstrated that intra-arterial cold saline infusion (IA-CSI) significantly reduces infarct volume and suppresses reperfusion injury by selectively lowering the temperature of ischemic brain tissue, with mechanisms involving inhibition of inflammatory responses, preservation of blood–brain barrier integrity, and improvement of microcirculation. Rodent and large animal experiments confirmed that IA-CSI rapidly achieves localized hypothermia within 5–10 min without inducing systemic side effects [44,45,46,47,48]. Preliminary clinical studies have validated the safety and feasibility of this approach. A prospective cohort study involving 113 patients revealed that short-duration IA-CSI combined with mechanical thrombectomy (MT) significantly reduced final infarct volume (*p* = 0.038), though 90-day functional independence (modified Rankin Scale, mRS 0–2) only showed a trend toward improvement (51.1% vs. 41.2%, *p* = 0.192) [10]. Another small single-arm study (*n* = 26) demonstrated the safe implementation of IA-CSI combined with recanalization techniques, with estimated brain temperature reductions ≥2 °C and no electrolyte imbalances or hemodynamic fluctuations [50]. Current clinical evidence remains limited by small sample sizes, non-randomized designs, and lack of direct brain temperature monitoring. Key unresolved issues include optimization of saline infusion volume, duration of localized hypothermia, and synergistic effects with systemic cooling. Future research requires large-scale randomized controlled trials to validate therapeutic efficacy and explore novel technologies (e.g., insulated catheters to minimize thermal loss, autologous blood-based hypothermic circulation systems) to enhance cooling efficiency. Concurrently, integrating multimodal imaging to dynamically assess ischemic penumbra evolution will ultimately enable precise and individualized hypothermic neuroprotective strategies. Although IA-CSI achieves rapid and targeted cooling of ischemic brain tissue, its reliance on complex endovascular procedures limits broad clinical adoption. Subsequent innovations revealed that internal jugular vein cooling achieved comparable cerebral temperature reduction (33.9 °C within 30 min) while leveraging internal jugular vein catheterization—a bedside routine procedure—thereby markedly improving clinical feasibility. Animal experiments have confirmed that internal jugular vein cooling provides neuroprotective outcomes comparable to intracarotid hypothermia, marking a critical step toward the clinical translation of localized cooling technologies [51]. Clinical Studies of Hypothermia in Ischemic Stroke Patients and Volunteers is presented in Table 1.

### 3.3. Induced Hypothermia and Synergistic Drug Strategies: Mechanistic Insights and Precision Modulation

To further optimize the therapeutic efficacy of hypothermia, studies have demonstrated that combining hypothermia with pharmacological interventions exerts significant synergistic neuroprotective effects in ischemic stroke. As a novel hypothermic perfusate, magnesium sulfate (MgSO_4_) combined with hypothermia therapy was found in animal and in vitro cellular experiments to significantly improve neurological function, reduce infarct volume, and protect the integrity of neurovascular units by regulating calcium homeostasis. Compared to intra-arterial selective cooling saline infusion, MgSO_4_ combined with hypothermia therapy not only reverses the early inhibitory effect of hypothermia on cerebral blood flow but also enhances the protective effects on neurons and cerebral microvascular endothelial cells by inhibiting calcium influx and oxidative stress damage [53]. These findings indicate that MgSO_4_ as a hypothermic perfusate has significant neuroprotective potential and provides new strategies and molecular mechanisms support for future clinical treatments of ischemic stroke. Hong An et al. first demonstrated synergistic neuroprotection through combined mild hypothermia (33–35 °C) and phenothiazine neuroleptics (chlorpromazine + promethazine) in a rat reversible right middle cerebral artery occlusion (MCAO) model. This combinatorial intervention created a pro-survival cellular environment via coordinated upregulation of anti-apoptotic proteins (Bcl-2, Bcl-xL) and suppression of pro-apoptotic effectors (AIF, Bax). Crucially, the neuroprotective mechanism was shown to pivotally involve the PI3K/Akt pathway, as PI3K/Akt inhibitors completely abolished therapeutic efficacy, definitively establishing it as a central therapeutic target [54]. Future investigations should prioritize multi-omics integration (transcriptomics/proteomics) to map PI3K/Akt downstream interactomes and their crosstalk with complementary pathways (e.g., NF-κB). Jun Zhang et al. demonstrated that combined application of a low-dose TRPV1 agonist dihydrocapsaicin (DHC) and physical cooling with ice pads achieved rapid synergistic hypothermia in a rat ischemic stroke model, significantly outperforming monotherapies. The combination therapy exhibited synergistic enhancements in neurological deficits, cerebral infarct volume, oxidative metabolism and apoptotic regulation [41]. This multi-mechanistic strategy overcomes limitations of conventional hypothermia therapies, offering a promising, safe, and efficient potential therapeutic approach for neuroprotection in clinical ischemic stroke, while further exploration of its long-term efficacy and translational potential is warranted. Additionally, preclinical evidence supports that combining hypothermia with thrombolytic therapy (e.g., recombinant tissue plasminogen activator, rt-PA) [21] anti-inflammatory agents (e.g., minocycline) [22], antioxidants (e.g., edaravone) [23], neurotrophic factors (e.g., brain-derived neurotrophic factor, BDNF) [55], and decompressive procedures (e.g., decompressive craniectomy) [56] can significantly reduce infarct volume, improve neurological outcomes, mitigate reperfusion injury, and extend the therapeutic window. However, some clinical studies have failed to demonstrate significant advantages of combination therapy, which could be attributed to variations in treatment timing, hypothermia depth, and drug metabolism [57,58]. Future efforts should focus on optimizing combination strategy parameters (e.g., dosage, timing) and advancing the efficient clinical translation of this multimodal approach through multicenter randomized controlled trials.

### 3.4. Factors That Influence the Efficacy of Hypothermia

In the treatment of ischemic stroke, the efficacy of therapeutic hypothermia is influenced by multiple interrelated factors. First, treatment timing is critical; early initiation of hypothermia increases opportunities to “freeze” ischemic penumbra tissue, thereby improving clinical outcomes [40]. Second, optimization of therapeutic parameters—including cooling rate, target temperature depth, duration, and rewarming speed—is essential for maximizing neuroprotective effects. Rapid achievement of target temperature enhances cerebral protection and reduces ischemic damage [59]. The depth of hypothermia represents another key parameter. One study has indicated that mild hypothermia demonstrates superior neuroprotection compared to moderate hypothermia, with better tolerance and recovery in rats. However, deeper hypothermia may achieve more significant reductions in cerebral infarction volume, though these effects vary across experimental models and conditions [60]. Clinically, a balance must be struck between hypothermia depth and complications such as arrhythmias and hypotension, as excessive cooling may negate therapeutic benefits. Given the cumulative evidence from cardiology and the aforementioned clinical considerations, maintaining mild hypothermia with a temperature set at 33–34 °C appears to strike an optimal balance between efficacy and safety. Hypothermia duration directly correlates with neuroprotection. Since post-stroke injury cascades persist for hours to days, therapeutic hypothermia should theoretically span these harmful processes. Preclinical evidence suggests prolonged cooling enhances neuroprotection [61], yet extended durations increase risks of complications such as infections, potentially offsetting benefits. Therefore, it is essential to carefully balance these factors to maximize efficacy while minimizing adverse effects. Rewarming speed critically determines patient outcomes. Rapid rewarming may damage erythrocytes or coagulation factors, trigger rebound intracranial hypertension (potentially causing brain herniation or death), or induce hyperkalemia, which can precipitate malignant arrhythmias [62,63]. Thus, maintaining a slow rewarming speed is crucial. In clinical practice, a slow rewarming protocol over 12 h is recommended to ensure patient safety. Finally, vascular recanalization status and combination therapies significantly influence outcomes. Successful recanalization is pivotal for hypothermia’s neuroprotective efficacy, while synergistic use with thrombolysis or other neuroprotective agents may further enhance therapeutic benefits [64].

## 4. Remote Ischemic Conditioning

Remote Ischemic Conditioning (RIC) is a procedure that involves applying brief cycles of ischemia and reperfusion to a limb (such as an arm or leg), typically using a blood pressure cuff for intermittent inflation and deflation. This process activates endogenous protective mechanisms within the body, enhancing cerebral tolerance to ischemia, increasing local cerebral blood flow, promoting the establishment of collateral circulation, reducing cerebral hemorrhage, and alleviating inflammatory responses, thereby providing protection against cerebral ischemic injury [65,66,67,68,69,70]. In 1986, Murry et al. first discovered in cardiac research that ischemic preconditioning could protect myocardium from subsequent prolonged ischemic injury. Subsequently, in 1994, Przyklenk et al. proposed the concept of remote ischemic preconditioning, which involves applying transient ischemia to a limb distant from the target organ to protect the heart. This foundational work provided the theoretical basis for extending RIC to stroke. Since then, numerous animal experiments have investigated the protective mechanisms of RIC in cerebral ischemia, revealing its ability to mitigate ischemic brain injury through multiple pathways, including neural, humoral, and immune pathways [71,72,73]. The application of RIC in stroke has evolved progressively from preclinical research to clinical trials, demonstrating protective efficacy in both acute ischemic stroke and chronic cerebrovascular diseases.

### 4.1. Neuroprotective Mechanisms of Remote Ischemic Conditioning

RIC achieves neuroprotection in stroke through multidimensional mechanisms. In the humoral pathway, transient limb ischemia stimulates the release of circulating mediators such as miRNAs, SDF-1α, IL-10, nitric oxide/nitrite, which are transported via circulation to cerebral tissues, suppressing inflammatory responses and activating endogenous protective pathways [74,75,76,77]. In the neural pathway, RIC activates the autonomic nervous system (e.g., dorsal motor neurons of the vagus) and peripheral sensory afferent fibers, enhancing parasympathetic activity and modulating neural signaling [78,79,80]. In immunomodulation, RIC inhibits the release of pro-inflammatory cytokines (TNF-α, IL-6), reduces C-reactive protein (CRP) and leukocyte adhesion molecule expression, thereby mitigating post-ischemic neuroinflammation [71]. Hemodynamic optimization manifests as increased regional cerebral blood flow and enhanced collateral circulation formation [81,82,83,84]. Mitochondrial protection is mediated by activating ATP-sensitive potassium channels (KATP), inhibiting mitochondrial permeability transition pore (MPTP) opening, reducing ROS generation and cytochrome C release, thereby preserving energy metabolic homeostasis [85,86,87,88]. Additionally, RIC upregulates neurotrophic factors (e.g., VEGF, BDNF) [89,90,91,92,93], promoting neuroregeneration and synaptic plasticity. These mechanisms synergistically establish a cross-organ, multitarget neuroprotective network against acute and chronic cerebral ischemia.

### 4.2. Research Progress on Remote Ischemic Conditioning in Neuroprotection Against Stroke

#### 4.2.1. Preclinical Studies on RIC

RIC, as a non-invasive neuroprotective strategy, has demonstrated significant potential in preclinical studies in recent years. Multiple studies have shown that RIC alleviates ischemic brain injury and improves neurological functional recovery through various mechanisms. He et al. found that RIC significantly improved neurological deficits in rats treated with rtPA by reducing blood–brain barrier disruption, intracerebral hemorrhage, and cerebral infarction volume. The underlying mechanism may be associated with the decreased activation of platelet-derived growth factor receptor α (PDGFRα) in ischemic brain tissue and reduced levels of platelet-derived growth factor CC (PDGF-CC) in the blood [94]. Sun et al. further revealed that RIC mitigates oxidative stress and inflammatory responses by activating the Nrf2/HO-1 pathway, thereby improving the neurobehavioral function of MCAO reperfusion mice [95]. Additionally, Liu et al. discovered that RIC significantly altered the levels of various immune cell populations and circulating cytokines by modulating peripheral immune responses, thus reducing the inflammatory response after cerebral ischemia [71]. These studies not only confirmed the neuroprotective effects of RIC but also provided a theoretical basis for its clinical application. In terms of neural repair, RIC may enhance the neural repair process after ischemic brain injury through multiple mechanisms, including promoting neurogenesis, angiogenesis, and axonal regeneration. Li et al. found that limb remote ischemic conditioning (LRIC) promotes neurogenesis in ischemic mice by modulating the miR-449b/Notch1 pathway, thereby improving neurological function [96]. Ren et al. investigated the effects of LRIC on arteriogenesis and Notch signaling pathway activity in the brains of rats after ischemic stroke. The results showed that LRIC significantly increased local cerebral blood flow and promoted arteriogenesis in ischemic brain tissue, characterized by increased arterial diameter and proliferation of vascular smooth muscle cells. Moreover, LRIC treatment upregulated the expression of Notch1 and its intracellular domain (NICD) in arteries surrounding the ischemic area. These findings suggest that the therapeutic effects of LRIC may involve promoting arteriogenesis during the recovery period of focal cerebral ischemia, with the Notch1 signaling pathway playing an important role in LRIC-mediated arteriogenesis [97]. Therefore, RIC not only has neuroprotective effects in the acute phase but may also play an important role in the long-term recovery after ischemic brain injury.

Despite the encouraging results of RIC in preclinical studies, there are still many challenges in its clinical application. Future research needs to further explore the optimal timing and duration of RIC implementation to determine its efficacy in different stages (acute, subacute, and chronic). In addition, given the regulatory effects of RIC on the immune system, future studies should delve into its long-term impact on the central nervous system and peripheral immune system, as well as whether this regulation can be translated into long-term neurological functional improvement. With a deeper understanding of the mechanisms of RIC and its dual roles in neuroprotection and neural repair, RIC is expected to become a safe and effective therapeutic strategy for ischemic brain injury, providing new ideas and methods for clinical practice.

#### 4.2.2. Clinical Studies on RIC

Recent randomized controlled trials (RCTs) exploring the efficacy of RIC across diverse stroke subgroups have yielded heterogeneous outcomes (Table 2).

RESCUE BRAIN Trial enrolled 188 patients with carotid artery ischemic stroke confirmed by magnetic resonance imaging (MRI) within 6 h of symptom onset. RIC applied during or after reperfusion therapy failed to significantly reduce 24-h infarct growth or improve clinical outcomes. This may relate to delayed timing (post-reperfusion) or insufficient intervention intensity. However, safety was affirmed despite 52.6% of patients experiencing cuff-related pain [98].

The RESIST trial included 1500 patients with acute stroke (ischemic and hemorrhagic). RIC treatment initiated in the prehospital setting and continued in the hospital for 7 days did not significantly improve functional outcomes at 90 days (*p* = 0.67) and did not increase the incidence of serious adverse events, suggesting limited benefits of RIC in acute stroke treatment [99]. In a post hoc subgroup analysis of the RESIST trial focusing on acute ischemic stroke subtypes, researchers found that for patients with acute ischemic stroke due to small vessel disease, if good treatment adherence (completion of at least 80% of the planned RIC cycles) could be maintained, RIC treatment was significantly associated with improved functional outcomes at 90 days compared with sham treatment (*p* = 0.013). However, no significant association was found between RIC treatment and functional outcomes for other stroke subtypes [100]. These findings are hypothesis-generating for future trials.

The RICAMIS trial focused on patients with moderate ischemic stroke (NIHSS 6–16). High-intensity RIC (200 mmHg) applied to both upper limbs twice daily for 10 to 14 days combined with standard care significantly improved the likelihood of favorable outcomes at 90 days (*p* = 0.02) This suggests that long-term RIC may improve long-term outcomes in patients with specific stroke severity through neurorestorative mechanisms such as angiogenesis [101]. To further investigate whether the duration of long-term remote ischemic conditioning after stroke is associated with better clinical outcomes in ischemic stroke, a post hoc analysis of the RICAMIS trial was conducted in patients with acute moderate ischemic stroke. The results showed that compared with the control group who did not receive RIC, patients who received RIC for 11 to 13 days had a significantly higher proportion of good functional outcomes at 90 days (*p* = 0.001), which may be related to the promotion of angiogenesis and neurorestoration by long-term RIC. However, patients who received RIC for 1 to 7 days had a lower proportion of good functional outcomes compared with the control group (*p* = 0.05), which may be related to early neurological deterioration leading to treatment discontinuation. In terms of safety, there were no significant differences in adverse events between the groups [102].

The SERIC-IVT trial evaluated the efficacy of RIC in combination with intravenous thrombolysis (IVT) and found no significant improvement in 90-day functional outcomes (*p* = 0.17) despite favorable safety (*p* = 0.22). This may be related to the relatively mild symptoms of the enrolled patients (median NIHSS 6) and the delayed initiation of RIC (median 13.5 h) [103].

The RICA trial targeted patients with symptomatic intracranial atherosclerotic stenosis (ICAS) and found that daily RIC treatment did not reduce the risk of ischemic stroke in the overall population after a median follow-up of 3.5 years (*p* = 0.12). However, in the subgroup with adherence ≥50%, RIC was significantly effective (*p* = 0.038), and the risk of the composite endpoint (stroke/TIA/myocardial infarction) was reduced by 18% (*p* = 0.0089), highlighting the critical role of long-term compliance [104].

In summary, regarding the timing and intensity of treatment, RIC has limited efficacy in the acute phase (as seen in the RESCUE BRAIN, RESIST, and SERIC-IVT trials), while it may be more effective in moderate to severe stroke (RICAMIS trial) or long-term treatment (RICA trial). In terms of population specificity, RIC appears to be more targeted at patients with moderate ischemic stroke (RICAMIS trial) and ICAS patients with high adherence (RICA trial), and no synergistic effect was observed when combined with reperfusion therapies such as IVT. From the perspective of adherence, the RICA trial indicates that efficacy is closely related to the continuity of treatment, suggesting that future interventions need to optimize strategies (such as simplifying procedures and portable devices) to improve long-term patient adherence. These findings inform precision RIC application. Future studies must clarify optimal intervention windows, dosing, and mechanistic interplay with reperfusion therapies.

**Table 2:** Clinical Studies of RIC in Ischemic Cerebrovascular Disease.

Study	Study Type	Sample Size	Type of Patients	Treatment	Primary Outcome	Main Results
RESCUE BRAIN (NCT02189928)	multi-center prospective RCT	188	Patients of AIS within 6 h of symptom onset	4 cycles of 5 min of cuff inflation and deflation on the thigh of the unaffected side Cuff pressure: 110 mmHg above systolic pressure Times: Once prehospital	Brain MRI changes of DWI brain infarction volume between baseline and day 1	RIC cannot limit brain infarction volume growth at 24 h after symptom onset.
RESIST (NCT03481777 )	multicenter prospective RCT	1500	patients with prehospital stroke symptoms for less than 4 h	5 cycles of 5 min of cuff inflation and deflation on 1 upper extremity Cuff pressure: 200 mm Hg or 35 mmHg higher than the systolic blood pressure Times: once prehospital and twice daily for 7 days in hospital	mRS at 3 months	RIC did not significantly improve functional outcome at 90 days among patients with acute stroke.
RICAMIS (NCT03740971)	multicenter prospective RCT	1893	patients with acute moderate ischemic stroke	5 cycles of cuff inflation for 5 min and deflation for 5 min to the bilateral upper limbs Cuff pressure: 200 mm Hg Times: within 48 h after symptom onset; twice daily for 10 to 14 days	mRS at 3 months	RIC was safe and significantly increased the likelihood of excellent neurologic function at 90 days.
RICA (NCT02534545)	multicenter prospective RCT	3033	patients aged 40–80 years with ischemic stroke or transient ischemic attack attributable to angiographically verified 50–99% stenosis of a major intracranial artery	5 cycles of cuff inflation for 5 min and deflation for 5 min to the bilateral upper limbs Cuff pressure: 200 mm Hg Times: once per day for the first 12 months after randomization	The time from randomization to the first occurrence of fatal or non-fatal ischemic stroke.	RIC did not reduce the risk of ischemic stroke in patients with symptomatic ICAS.
SERIC-IVT (NCT04980625)	multicenter prospective RCT	558	patients with acute ischemic stroke who underwent IVT	5 cycles of cuff inflation for 5 min and deflation for 5 min to the unilateral upper limb of the unaffected side Cuff pressure: 200 mm Hg Times: twice daily for 7 days	mRS at 3 months	RIC was safe in patients with acute ischemic stroke who received IVT. However, it did not significantly improve excellent functional outcome.

AIS, Acute Ischemic Stroke; RCT, Randomized Clinical Trial; RIC, Remote Ischemic Conditioning; MRI, Magnetic Resonance Imaging; DWI, Diffusion Weighted Imaging; mRS, modified Rankin Score; IVT, Intravenous Thrombolysis; ICAS, Intracranial Atherosclerotic Stenosis.

## 5. Normobaric Hyperoxia

Normobaric hyperoxia (NBO) refers to the therapeutic approach of inhaling high concentrations of oxygen (typically ranging from 40% to 100%) under normal atmospheric pressure to enhance tissue oxygenation levels [105,106,107]. One of the core mechanisms underlying ischemic brain injury is the hypoxic state of brain tissue. In recent years, the application of NBO in AIS has garnered widespread attention. As a non-invasive and technically straightforward intervention, NBO demonstrates neuroprotective potential by improving cerebral oxygenation levels, preserving blood–brain barrier integrity, enhancing mitochondrial function, reducing oxidative stress, and suppressing apoptosis, thereby potentially improving outcomes in ischemic brain injury [108].

### 5.1. Mechanisms of NBO

#### 5.1.1. Increasing Oxygenation of Brain Tissue

Li et al. demonstrated that maintaining the oxygenation state of the ischemic penumbra through NBO treatment during ischemia significantly reduced infarct volume and improved neurological function, thereby exerting neuroprotective effects. The mechanisms may involve reducing the production of ROS, inhibiting the activation of matrix metalloproteinase-9 (MMP-9), and caspase-8. This study also revealed that the impact of NBO on cerebral blood flow (CBF) varies depending on the timing of treatment: administration during ischemia increases CBF in the penumbra, whereas administration during reperfusion decreases it [109]. Additionally, Shin et al. found that NBO treatment provides neuroprotection against ischemic brain injury by enhancing tissue oxygenation, improving CBF, and inhibiting peri-infarct depolarizations (PIDs) [110].

#### 5.1.2. Protecting the Integrity of the Blood–Brain Barrier (BBB)

Ischemia/reperfusion-induced BBB damage is closely associated with hemorrhagic transformation following vascular recanalization. Therefore, maintaining the integrity of the BBB during ischemia may play a crucial role in reducing hemorrhagic transformation and improving the overall prognosis of stroke patients [111]. Liang et al. discovered that NBO could slow down the progression of ischemia-induced BBB damage, as evidenced by reduced Evans blue leakage, brain edema, and hemorrhagic volume. NBO protects the BBB by inhibiting MMP-9 induction and the loss of tight junction proteins such as occludin and claudin-5. Even when tPA is administered after prolonged ischemia (up to 7 h), NBO combined with tPA treatment still significantly improves neurological function, reduces brain edema, hemorrhagic volume, and mortality [112]. This provides new evidence supporting NBO as an adjunctive therapy to tPA thrombolysis to extend the therapeutic time window. Clinical trials conducted by Li et al. showed that at 24 h and 7 days, serum levels of occludin and MMP-9, markers of BBB damage, were significantly lower in the NBO combined with endovascular treatment (EVT) group compared to the EVT-alone group. Moreover, the incidence of intracranial contrast agent extravasation at 24 h was significantly reduced (35.9% vs. 60.5%). Furthermore, serum occludin levels at 7 days were identified as an independent predictor of poor prognosis at 90 days [113]. Both clinical and animal experimental studies have confirmed the potential of NBO in protecting the integrity of the BBB, reducing brain injury, and improving the therapeutic outcomes of ischemic stroke.

#### 5.1.3. Improving Mitochondrial Function

Mitochondrial dysfunction can occur following ischemic brain injury, leading to oxidative stress and energy depletion, which in turn result in neuronal apoptosis and necrosis [114]. Studies have shown that NBO significantly reduces the number of zinc-staining positive cells and zinc-staining intensity in penumbral tissues, but not in the ischemic core. Moreover, NBO or the zinc-specific chelator TPEN can significantly attenuate ischemia-induced zinc accumulation in mitochondria, stabilize the mitochondrial membrane potential in the penumbra after cerebral ischemia, and significantly reduce ischemia-induced cytochrome C release from mitochondria in penumbral tissues [115]. These results uncover a novel mechanism of NBO’s neuroprotective effects, specifically in reducing ischemia-induced mitochondrial damage in penumbral tissues by decreasing mitochondrial zinc accumulation, providing further evidence for the potential clinical application of NBO in the treatment of acute ischemic stroke.

#### 5.1.4. Reducing Oxidative Stress and Inhibiting Apoptosis

The study by Tang et al. demonstrated that NBO treatment significantly elevated tissue oxygen partial pressure (pO2) in the ischemic penumbra to levels approaching pre-ischemic values, while reducing MRI apparent diffusion coefficient lesion volume, indicating neuroprotective effects. Mechanistic investigations revealed that cerebral ischemia–reperfusion upregulated the mRNA and protein expression of gp91phox, the catalytic subunit of NADPH oxidase, whereas NBO treatment markedly suppressed this upregulation and diminished NADPH oxidase activity, thereby attenuating ROS generation. The study confirmed that NBO alleviates oxidative damage by inhibiting NADPH oxidase-dependent ROS production [116]. These findings provide a theoretical foundation for the clinical application of NBO as a safe and rapid neuroprotective strategy, highlighting its therapeutic efficacy through modulation of oxidative stress pathways without exacerbating oxygen free radical injury. Jin et al. demonstrated that NBO combined with minocycline exerts neuroprotective effects in transient focal cerebral ischemia. The combination therapy not only inhibited the induction of matrix metalloproteinases (MMP-2/9) and their mediated degradation of the tight junction protein occludin, thereby preserving BBB integrity, but also reduced the activation of caspase-3/9 and the expression of apoptosis-inducing factor (AIF), simultaneously blocking both caspase-dependent and -independent apoptotic pathways [117]. This combination regimen offers time-sensitive advantages and safety, providing experimental evidence for a multi-target therapeutic approach in ischemic stroke and proposing a novel strategy for its treatment.

Collectively, these research findings support the neuroprotective potential of NBO in ischemic brain injury, highlight the importance of early intervention, and provide a scientific basis for future clinical applications.

### 5.2. Advances in Clinical Research on NBO

#### 5.2.1. NBO Monotherapy

In 2012, Wu et al. evaluated the efficacy of NBO in acute ischemic stroke using MRI predictive models. Results showed that during NBO treatment (4 h), the predicted lesion volume in the control group was significantly larger than that in the NBO group (*p* = 0.007), indicating that NBO transiently slowed ischemic lesion expansion. However, no significant differences were observed post-treatment or at discharge [118]. This study suggested that NBO may exert neuroprotective effects by extending the thrombolytic time window and improving cerebral blood flow, highlighting its therapeutic potential in acute stroke (Table 3).

#### 5.2.2. Combination of NBO with Reperfusion Therapy

While reperfusion is an effective treatment for AIS, patient outcomes remain suboptimal. Clinical studies have demonstrated enhanced efficacy when combining NBO with reperfusion strategies (Table 3).

##### NBO Combined with Intravenous Thrombolysis

The combination of NBO and intravenous thrombolysis significantly improves neurological function scores, reduces infarct volume, and lowers the risk of hemorrhagic transformation in AIS patients. In 2017, Shi et al. investigated the impact of NBO on blood tight junction protein Occludin in AIS rats and patients. They found that blood Occludin dynamically reflects BBB damage, and NBO attenuates BBB disruption by inhibiting Occludin degradation, thereby improving neurological recovery post-ischemia–reperfusion. Clinical data revealed significantly reduced blood Occludin levels and faster functional recovery in NBO-treated AIS patients, suggesting blood Occludin as a potential biomarker for BBB integrity [119]. However, the small sample size necessitates further validation. Although cohort studies provided important prognostic data over extended follow-up periods, sufficiently powered RCTs are still necessary to confirm causal relationships. In 2021, a single-center observational cohort study evaluated the efficacy and safety of NBO combined with intravenous thrombolysis in 227 AIS patients with anterior circulation stroke treated within 4.5 h. Results demonstrated that NBO combined with thrombolysis significantly improved 90-day functional outcomes (*p* = 0.002) and reduced infarct volume (*p* = 0.006). Multivariate analysis confirmed NBO combination therapy as an independent predictor of functional independence (*p* = 0.01), with no significant differences in intracranial hemorrhage or mortality [122]. NBO may synergize with thrombolysis by enhancing penumbral oxygenation and delaying cell death, though prospective studies are needed to confirm its clinical value.

##### NBO Combined with Endovascular Therapy (EVT)

NBO combined with EVT has also shown promise in improving AIS outcomes. In 2021, a prospective randomized controlled trial evaluated the safety and efficacy of adjuvant high-flow NBO (FiO_2_ 50%, 15 L/min) following MT for anterior circulation large vessel occlusion. Compared to conventional low-flow oxygen therapy (3 L/min), the NBO group exhibited superior 90-day mRS scores, reduced mortality, smaller infarct volumes, and no increase in symptomatic intracranial hemorrhage or pneumonia [120]. This study highlights NBO’s neuroprotective potential in reperfusion therapy. In 2022, the OPENS-1 trial by Li et al. assessed NBO combined with EVT in acute anterior circulation large vessel occlusion. Results showed that the NBO + EVT group had significantly smaller 24–48-h MRI infarct volumes (*p* < 0.01) and better 90-day mRS scores (*p* = 0.038) than the EVT group, without increasing complications like symptomatic intracranial hemorrhage or mortality [9]. This study supports NBO’s synergistic role in post-reperfusion neuroprotection, though its single-center design and limited sample size warrant further validation. The OPENS-2 trial, a multicenter, randomized, single-blind, sham-controlled study across 26 Chinese stroke centers, evaluated 100% NBO (10 L/min for 4 h) combined with EVT in 282 patients. The NBO + EVT group showed significantly improved 90-day mRS scores with no increase in mortality or adverse events, confirming its safety and efficacy [123]. These findings suggest that NBO stabilizes the ischemic penumbra and inhibits post-reperfusion neuronal apoptosis.

### 5.3. Factors Influencing NBO Efficacy

Despite NBO’s potential, optimal treatment protocols require further investigation. Current evidence indicates that treatment duration and oxygen concentration significantly impact outcomes. Henninger et al. demonstrated that NBO reduced infarct volume by 44% in permanent MCAO when administered for 6 h [126]. A single-center dose-escalation study found that 4–6 h of NBO effectively reduced infarct volume and improved neurological function in EVT patients, with 4 h recommended as the optimal balance between efficacy and tolerability [125]. However, larger multicenter trials are needed to validate these findings. Regarding oxygen concentration, Chen et al. proposed intermittent high-concentration (50–90%) short-duration protocols combined with antioxidants to minimize adverse effects [127]. Prolonged use of 100% oxygen may exacerbate excitotoxicity or cerebral edema. Thus, clinical translation requires optimization of parameters and validation through randomized controlled trials to ensure long-term safety and efficacy.

NBO, as a simple and non-invasive therapy, demonstrates significant neuroprotective effects in AIS. Future research should focus on multicenter randomized controlled trials and neuroimaging studies to refine treatment protocols, validate efficacy, and ensure safety, ultimately providing more effective therapeutic options for AIS patients.

## 6. Conclusions

Non-pharmacological neuroprotective strategies for AIS are biologically rational. Pre-clinical work indicates that RIC, NBO and mild hypothermia can mitigate the progression of the ischemic penumbra, reducing infarct volume by inhibiting apoptosis, attenuating inflammatory responses, preserving BBB integrity, promoting neural repair, and enhancing collateral circulation. In clinical trials, non-pharmacological neuroprotective therapies have demonstrated certain safety, good tolerability, and low cost. However, their clinical value remains in a state of “mixed evidence,” and several challenges persist in their application. Future research should focus on the following: (i) identifying the optimal combination of intervention timing, dosage, treatment duration, and reperfusion strategies; (ii) utilizing imaging-biomarkers to identify potential beneficiary subpopulations; and (iii) exploring synergistic mechanisms between non-pharmacological and pharmacological neuroprotection to advance the development of truly translatable, precision neuroprotective strategies. In summary, non-pharmacological neuroprotective therapies are expected to become an important component of comprehensive treatment strategies for ischemic stroke, offering patients additional therapeutic options through innovative, accessible, and cost-effective approaches.

## Figures and Tables

**Table 1:** Clinical Studies of Hypothermia in Ischemic Stroke Patients and Volunteers.

Study	Study Type	Sample Size	Type of Patients	Treatment	Main Results
L Covaciu et al. (2011) [52]	prospective, nonrandomized and single arm study	10	awake volunteers	Intranasal balloons catheters circulated with saline at 20 °C were applied for 60 min	Brain temperature decreased in volunteers subjected to intranasal cooling.
Chen et al. (2016) [50]	nonrandomized and single arm observational study	26	patients with LVO within 8 h after symptom onset	50 mL cold 0.9% sodium chloride (4 °C) into the ischemic territory at 10 mL/min before recanalization, 30 mL/minute for 10 min as soon as blood flow was restored	Intra-arterial hypothermia with endovascular recanalization therapy in AIS appears feasible and safe.
Wu et al. (2018) [10]	prospective non-RCT	113	patients with LVO-induced AIS receiving MT	IA-SCI with 350 mL 0.9% saline at 4 °C for 15 min pre- and post-reperfusion	Combining short-duration IA-SCI with MT was safe. IA-CSI was effective in reducing infarct volume but cannot improve the functional outcomes at 90 days.

LVO, Large Vessel Occlusion; RCT, Randomized Clinical Trial; AIS, Acute Ischemic Stroke; IA-SCI, Intraarterial Selective Cooling Infusion; MT, Mechanical Thrombectomy.

**Table 3:** NBO Monotherapy and combined with Reperfusion Strategy.

Study Sponsor	Year	Study Type	Sample Size	Type of Patients	NBO Treatment and Duration	Primary Outcome	Results
NBO Monotherapy							
Ona Wu et al. [118]	2012	Prospective RCT	16	AIS within 12 h of onset or <15 h after last seen neurologically intact with PWI/DWI mismatch >20% NBO group: *n *= 10, Control group: *n* = 6	High-flow oxygen via facemask for 8 h	Lesion volume change (4 h, 24 h and discharge)	NBO significantly attenuated ischemic lesion growth during therapy (4 h). No significant differences in lesion volumes at later time points (24 h and discharge).
Combination of NBO with Reperfusion Therapy							
Shi et al. [119] (NCT02974283)	2017	Cohort Study	18	AIS patients receiving IVT NBO group: *n* = 9, Normoxia group: *n* = 9	Oxygen, a flow rate of 10 L/min, 4 h, by facemask	Blood Occludin and Claudin-5 Levels (at admission, 24 h, 72 h) NIHSS (at admission, 24 h, 72 h and 1 week)	NBO reduced blood occludin and improved neurological functions in AIS patients.
Cheng et al. [120] (ChiCTR-INR-17013685)	2017	Prospective RCT	175	AIS patients with anterior circulation LVO receiving MT NBO group: *n* = 88, Control group: *n* = 87	FiO_2_ 50%, flow 15 L/min by Venturi mask for 6 h after MT	mRS at 90 days	High-flow NBO therapy after MT is safe and effective in improving functional outcomes.
Li et al. [9] (OPENS-1) (NCT03620370)	2018	Single-center, assessor-blinded RCT	86	AIS patients who received EVT NBO + EVT group: *n* = 43, EVT group: *n* = 43	Oxygen, 10 L/min for 4 h via facemask initiated before vascular recanalization	Cerebral infarct volume within 24–48 h after randomization	NBO in combination with EVT could significantly reduce infarct volume and enhance clinical outcomes at 90 days.
Poli et al. (PROOF trial) [121] (NCT03500939)	2019	Multi-center RCT	456	AIS patients due to anterior-circulation LVO likely to receive EVT Treatment group: Control group (1:1)	≥40 L/min oxygen via non-rebreather mask (FiO_2_ ≈100%) or ventilator (FiO_2_ 1.0) continued until MT completion or for 4 h if MT was not attempted/stopped	Ischemic core growth from baseline to 24 h	Terminated
Li na et al. [122]	2021	Single-center observational cohort study	227	AIS patients with anterior circulation LVO receiving IVT NBO group: *n* = 125, Control group: *n* = 102	Oxygen, 10 L/min for 4 h via facemask at the beginning of IVT	mRS at 90 days	Better prognosis in the NBO group.
Li et al. [123] (OPENS-2) (NCT04681651)	2021	Multi-center, single-blind, RCT	282	AIS patients with anterior circulation LVO within 6 h, who were candidates for endovascular treatment. NBO group: *n* = 140, Sham normobaric hyperoxia group: *n* = 142	100% oxygen at a flow rate of 10 L/min through a non-rebreather mask for 4 h	mRS at 90 days	Normobaric hyperoxia combined with endovascular treatment significantly improved 90-day functional outcome in AIS patients.
Wei et al. (OPENS-2L) [124] (NCT05039697)	2021	Multi-center, double-blind, RCT	282	AIS patients with anterior circulation LVO within 6 h, who were candidates for endovascular treatment. NBO group: *n* = 140, Sham normobaric hyperoxia group: *n* = 142	100% oxygen at a flow rate of 10 L/min through a non-rebreather mask for 4 h	mRS at 1 year	Completed
Li et al. (TD-NBO) [125] (NCT05404373)	2022	Single-center, single-blind, RCT	100	AIS patients who had an indication for endovascular treatment NBO group (2 h, *n* = 25; 4 h, *n* = 25; 6 h, *n* = 25); Low flow oxygen group (*n* = 25)	100% oxygen at a ventilation rate of 10 L/min using an oxygen storage mask for 2 h, 4 h and 6 h	The infarct volume within 72 h after randomization	NBO therapy for 4 and 6 h was found to be more effective.
Ji et al. (OPENS-3) (NCT05965687)	2023	Multi-center, single-blind, RCT	1230	AIS patients who undergo IVT within 4.5 h from onset NBO group: NBO+ rt-PA; Control group: Nasal oxygen+ rt-PA	100% oxygen at a ventilation rate of 10 L/min using a sealed non-ventilating oxygen storage mask for 4 h	Utility-weighted mRS at 90 days	On going
Ji et al. (OPENS-3L) (NCT05965193)	2023	Multi-center, single-blind, RCT	1230	AIS patients who undergo IVT within 4.5 h from onset NBO group: NBO+ rt-PA; Control group: Nasal oxygen+ rt-PA	100% oxygen at a ventilation rate of 10 L/min using a sealed non-ventilating oxygen storage mask for 4 h	Utility-weighted mRS at 12 months	On going
Ji et al. (AN-O2-Trans) (NCT06666764)	2024	Multi-center, single-blind, RCT	1500	AIS patients due to anterior-circulation LVO likely to receive EVT NBO group: NBO+ best medical practice, Control group: best medical practice	100% oxygen	mRS at 90 days	On going
Ji et al. (AN-O2-EMS) (NCT06801457)	2025	Multi-center, single-blind, RCT	1230	Patients with suspected AIS due to LVO presenting within 6 h of symptom onset NBO group: NBO+ best medical practice, Control group: best medical practice	100% oxygen	Utility-weighted mRS at 90 days	On going

Abbreviations: NBO, Normobaric Hyperoxia; RCT, Randomized Clinical Trial; mRS, modified Rankin Score; MRI, Magnetic Resonance Imaging; DWI, Diffusion Weighted Imaging; PWI, Perfusion Weighted Imaging; AIS, Acute Ischemic Stroke; IVT, Intravenous Thrombolysis; LVO, Large Vessel Occlusion; CT, computerized tomography; EVT, Endovascular Thrombectomy; FiO_2_, Fraction of Inspired Oxygen; MT, Mechanical Thrombectomy. Penumbral Rescue by Normobaric O2 Administration in Patients with Ischemic Stroke and Target Mismatch ProFile (PROOF). Normobaric Hyperoxia Combined with Reperfusion for Acute Ischemic Stroke (OPENS-1). Normobaric Hyperoxia Combined with Endovascular Treatment in Acute Ischemic Stroke (OPENS-2). Normobaric Hyperoxia Combined with Endovascular Therapy in Patients With Stroke Within 6 Hours of Onset: Longterm Outcome (OPENS-2L). Treatment Duration on Normobaric Hyperoxia in Acute Ischemic Stroke (TD-NBO). Normobaric Hyperoxia Combined with Intravenous Thrombolysis for Acute Ischemic Stroke (OPENS-3). Normobaric Hyperoxia Combined With Intravenous Thrombolysis for Acute Ischemic Stroke: Longterm Outcome (OPENS-3L). Ji et al. Normobaric Oxygen in AIS Transferred for EVT (AN-O2-Trans). Adjuvant Cerebroprotection Using Normobaric Hyperoxia in Pre-hospital Patients with Suspected Stroke (AN-O2-EMS).

## Data Availability

Not applicable.

## Abbreviations

AIS	Acute Ischemic Stroke
RIC	Remote Ischemic Conditioning
NBO	Normobaric Hyperoxia
FDA	Food and Drug Administration
FAS ligand	FASL
iASPP	inhibitor of Apoptosis-Stimulating Protein of p53
IACI	Intra-Arterial local Cooling
IA-CSI	Intra-Arterial Cold Saline Infusion
MT	Mechanical Thrombectomy
MgSO_4_	Magnesium Sulfate
rtPA	Recombinant Tissue-type Plasminogen Activator
MCAO	Middle Cerebral Artery Occlusion
LRIC	Limb Remote Ischemic Conditioning
IVT	Intravenous Thrombolysis
ICAS	Intracranial Atherosclerotic Stenosis
BBB	Blood–Brain Barrier
EVT	Endovascular Treatment
MRI	Magnetic Resonance Imaging
ROS	Reactive Oxygen Species
mRS	modified Rankin Scale
RCTs	Randomized Controlled Trials
MMP-9	Matrix Metalloproteinase-9
CBF	Cerebral Blood Flow
NF-κB	Nuclear Factor κB
